# Inhibition of metabotropic glutamate receptor 5 induces cellular stress through pertussis toxin-sensitive G_i_-proteins in murine BV-2 microglia cells

**DOI:** 10.1186/s12974-014-0190-7

**Published:** 2014-11-19

**Authors:** Boonrat Chantong, Denise V Kratschmar, Adam Lister, Alex Odermatt

**Affiliations:** Division of Molecular and Systems Toxicology, Department of Pharmaceutical Sciences, University of Basel, Klingelbergstrasse 50, 4056 Basel, Switzerland; Current address: Department of Preclinical Science and Applied Animal Science, Faculty of Veterinary Science, Mahidol University, Phutthamonthon, Nakhonpathom, Thailand

**Keywords:** Microglia, Glutamate receptor, mGluR5, Inflammation, Oxidative stress, Endoplasmic reticulum stress, Intracellular calcium

## Abstract

**Background:**

Activation of metabotropic glutamate receptor 5 (mGluR5) by (RS)-2-chloro-5-hydroxyphenylglycine (CHPG) was shown to suppress microglia activation and decrease the release of associated pro-inflammatory mediators. In contrast, the consequences of mGluR5 inhibition are less well understood. Here, we used BV-2 cells, retaining key characteristics of primary mouse microglia, to examine whether mGluR5 inhibition by 2-methyl-6-(phenylethynyl)-pyridine (MPEP) enhances cellular stress and production of inflammatory mediators.

**Methods:**

BV-2 cells were treated with MPEP, followed by determination of cellular stress using fluorescent dyes and high-content imaging. The expression of inflammatory mediators, endoplasmic reticulum (ER)-stress markers and phosphorylated AMPKα was analyzed by quantitative PCR, ELISA and Western blotting. Additionally, phospholipase C (PLC) activity, cellular ATP content and changes in intracellular free Ca^2+^ ([Ca^2+^]_i_) were measured using luminescence and fluorescence assays.

**Results:**

Treatment of BV-2 microglia with 100 μM MPEP increased intracellular reactive oxygen species (ROS), mitochondrial superoxide, mitochondrial mass as well as inducible nitric oxide synthase (iNOS) and IL-6 expression. Furthermore, MPEP reduced cellular ATP and induced AMPKα phosphorylation and the expression of the ER-stress markers CHOP, GRP78 and GRP96. The MPEP-dependent effects were preceded by a rapid concentration-dependent elevation of [Ca^2+^]_i_, following Ca^2+^ release from the ER, mainly via inositol triphosphate-induced receptors (IP_3_R). The MPEP-induced ER-stress could be blocked by pretreatment with the chemical chaperone 4-phenylbutyrate and the Ca^2+^ chelator BAPTA-AM. Pretreatment with the AMPK agonist AICAR partially abolished, whilst the inhibitor compound C potentiated, the MPEP-dependent ER-stress. Importantly, the PLC inhibitor U-73122 and the G_i_-protein inhibitor pertussis toxin (PTX) blocked the MPEP-induced increase in [Ca^2+^]_i_. Moreover, pretreatment of microglia with AICAR, BAPTA-AM, U-73122 and PTX prevented the MPEP-induced generation of oxidative stress and inflammatory mediators, further supporting a role for G_i_-protein-mediated activation of PLC.

**Conclusions:**

The results emphasize the potential pathophysiological role of mGluR5 antagonism in mediating oxidative stress, ER-stress and inflammation through a Ca^2+^-dependent pathway in microglia. The induction of cellular stress and inflammatory mediators involves PTX-sensitive G_i_-proteins and subsequent activation of PLC, IP_3_R and Ca^2+^ release from the ER.

**Electronic supplementary material:**

The online version of this article (doi:10.1186/s12974-014-0190-7) contains supplementary material, which is available to authorized users.

## Background

Neuroinflammation involves the activation and recruitment of immune cells, including microglia, macrophages and lymphocytes, as well as an expression of factors designed to respond to injury and aid in repair. Microglia are resident immune cells in the central nervous system and play an integral role in the neuroinflammatory response. However, excessive microglial activation is a hallmark of neurodegenerative diseases [1].

Metabotropic glutamate receptors (mGluRs) are expressed in many different cell types throughout the brain and spinal cord [2]. They have been considered as promising targets for neuro-protective agents in acute and chronic neurodegenerative disorders [3,4]. The mGluRs are G-protein-coupled receptors (GPCRs) consisting of eight subtypes divided into three groups (I to III) based on their sequence homology, signal transduction pathways and pharmacological profiles [3,5]. The mGluR5 belongs to the group I members, which are typically postsynaptic in neurons and mediate their signaling through Gαq-proteins. Activation results in the stimulation of phospholipase C (PLC) and phosphoinositide hydrolysis, leading to intracellular Ca^2+^ mobilization and activation of extracellular signal-regulated kinases 1 and 2 (ERK1/2) downstream signaling pathways [3]. High expression of mGluR5 was found in activated microglia [2,6], which surround the site of injury following traumatic brain injury in rats, suggesting pharmacological mGluR5 manipulation may be beneficial in neuroinflammatory diseases [7,8]. Indeed, the specific mGluR5 agonist (RS)-2-chloro-5-hydroxyphenylglycine (CHPG) was shown to inhibit microglial activation, oxidative stress and the release of inflammatory mediators both *in vitro* and *in vivo* [9–14]. Furthermore, the use of agonists to treat chronic spinal cord injuries has been proposed [4]. Thus, a dysregulation resulting in decreased mGluR5 activity may promote the initiation and/or progression of neurodegenerative disorders.

An excessive activation of microglia leads to the enhanced production of ROS and reactive nitrogen species (RNS), which promotes pro-inflammatory pathways via the activation of mitogen-activated protein kinases (MAPKs) and nuclear factor kappa-light-chain-enhancer of activated B cells (NF-κB) [15–18]. The mGluR5 affects ROS and nitric oxide (NO) production through inhibition of NADPH oxidase (NOX-2) activity [9,12–14]; however, the underlying mechanisms are not fully understood. The brain has a high energy demand and therefore depends on efficient mitochondrial function. Impaired mitochondrial dynamics and the chronic generation of ROS and RNS contribute to the pathogenesis of several neurodegenerative diseases [19,20]. A key regulator of mitochondrial adenosine triphosphate (ATP) production is AMP-dependent protein kinase (AMPK), which positively regulates signaling pathways replenishing ATP [21]. Although AMPK is considered to be a pro-survival kinase, its prolonged activation can induce endoplasmic reticulum (ER)-stress, thereby causing cell damage [22–25]. AMPK is directly targeted and activated by pro-oxidant species and by elevated intracellular Ca^2+^ levels ([Ca^2+^]_i_) [23,26], which can result in enhanced MAPK signaling and apoptosis.

Changes in [Ca^2+]^i have been implicated in the regulation of several activities of microglia, including proliferation, migration, cytokine release and ROS generation [27–30]. Different stimuli can lead to increased [Ca^2+^]i, either by Ca^2+^ release from the ER or by entry through the plasma membrane [31]. The ER Ca^2+^ store is regulated by two Ca^2+^ release channels, the inositol triphosphate-induced receptor (IP_3_R) [32,33] and the ryanodine receptor (RyR) [33], as well as by Ca^2+^ ATPases, which control Ca^2+^ reuptake into the ER [33]. Both, a prolonged Ca^2+^ depletion in the ER and a Ca^2+^ overload in the cytoplasm can cause ER-stress [34]. Activation of the unfolded protein response (UPR), consisting of the up-regulation of ER-chaperones, attenuation of protein translation and ER-associated degradation of misfolded proteins [35], counteracts ER-stress. The ER-resident molecular chaperones glucose-regulated protein 78 (GRP78) and glucose-regulated protein 94 (GRP94) as well as the transcription factor C/EBP homologous protein (CHOP) are induced by the UPR and they increase ER protein-folding capacity and maintain ER Ca^2+^ stores [35]. Prolonged ER-stress ultimately results in cell death by the apoptotic pathway mediated by caspase-12, an ER localized cysteine protease [35]. In addition to Ca^2+^ homeostasis, impaired mitochondrial function and AMPK signaling modulate the ER-stress response [36–38].

GPCRs play an important role in controlling microglial cell activity. The activation of mGluR5 in microglia has been suggested to involve the Gαq-protein signal transduction pathway through PLC, PKC and Ca^2+^ [10]. Upon stimulation of GPCRs, the inactive Gα-GDP/Gβγ heterotrimers release GDP and bind GTP, resulting in the dissociation of Gα from Gβγ. The resulting GTP-Gαq complex activates the β-isoforms of PLC [39]. However, a role for pertussis toxin (PTX)-sensitive Gi proteins in IP_3_ signaling and [Ca^2+^]_i_ increase has not, to our knowledge, been reported in microglia.

In order to elucidate the effects of the pharmacological inhibition of mGluR5 on cellular stress and inflammatory mediators in microglia, we tested the responsiveness of BV-2 mouse microglia to the selective mGluR5 antagonist MPEP. There is a close resemblance between BV2 cells and primary mouse microglia in terms of their inflammatory signaling pathways; therefore, BV-2 microglial cells can be used as a model for the activation of microglia *in vitro* [40,41]. In the present study, we show that BV-2 microglia represent a suitable cell model for studying the signaling pathway downstream of mGluR5. Our results provide evidence for a PLC- and IP_3_R-dependent increase in [Ca^2+^]_i_ following pharmacological mGluR5 inhibition. Importantly, the mGluR5-dependent effects could be blocked by PTX, indicating the involvement of a Gi protein-dependent signal transduction pathway.

## Experimental procedures

### Materials

DMEM, RPMI-1640 medium, penicillin, streptomycin, 0.05% (w/v) trypsin/EDTA, non-essential amino acids (NEAA), 4-(2-hydroxyethyl)-1-piperazineethanesulfonic acid (HEPES), Hank’s balanced salt solution (HBSS), EnzChek Direct PLC assay kit, Hoechst-33342, dihydroethidium (DHE), Mitotracker Red CMXRos, MitoSOX Red, Fluo-4 AM and probenecid were obtained from Invitrogen (Carlsbad, CA, USA). FBS was obtained from Connectorate (Dietikon, Switzerland), Cell Titer Glo® assay kit from Promega (Madison, WI, USA) and thapsigargin, AICAR, (RS)-2-chloro-5-hydroxyphenylglycine (CHPG), 6-methyl-2-(phenylethynyl)-pyridine (MPEP), BAPTA-AM, pertussis toxin (PTX), dantrolene sodium and sodium phenylbutyrate (PBA) from Sigma-Aldrich (St. Louis, MO, USA). Compound C and xestospongin C were obtained from Calbiochem (San Diego, CA, USA). Specific antibodies against GRP78, AMPK, and its phosphorylated form were obtained from Cell Signaling Technology (Danvers, MA, USA). Specific antibodies against β-actin and goat anti-rabbit IgG-horseradish peroxidase were obtained from Santa Cruz Biotechnology (Santa Cruz, CA, USA).

### Cell culture and experimental procedure

Murine BV-2 microglia, immortalized by infection with v-raf/c-myc recombinant retrovirus [42], were kindly provided by Professor Wolfgang Sattler, University of Graz, Austria. BV-2 cells were propagated in 75 cm^2^ flasks in RPMI-1640 medium supplemented with 10% FBS, 2 mM glutamine, 100 μg/mL streptomycin and 100 U/mL penicillin, 10 mM HEPES and 0.1 mM NEAA at 37°C, 5% CO_2_. Prior to initiating the experiments, cells were resuspended in DMEM supplemented with 10% FBS, 1 g/L glucose, 2 mM glutamine, 100 μg/mL streptomycin and 100 U/mL penicillin, 10 mM HEPES and 0.1 mM NEAA, and seeded again to reach 70% confluence. Cells were then distributed to 96-well (1 × 10^3^ cells/well), 24-well (2 × 10^4^ cells/well), 12-well (5 × 10^5^ cells/well) or 6-well (1.0 × 10^6^ cells/well) plates. Cells in the exponential growth phase (60 to 70% confluence) were used for the experiments.

### Determination of mRNA expression

Tri-reagent was used to isolate total RNA according to the manufacturer’s instructions (Sigma-Aldrich, St. Louis, MO, USA). Complementary DNA (cDNA) was synthesized from 0.5 μg total RNA using Superscript III reverse transcriptase (Invitrogen, Carlsbad, CA, USA). Briefly, in the first step 0.5 μg of sample RNA and 0.5 μg oligo (dT)15 were made up to a final volume of 12 μL in DNase/RNase-free water in 0.5 mL tubes, which were incubated at 65°C for 5 minutes and immediately transferred to ice. Secondly, reverse transcription reaction mixture (8 μL), containing 4 μL of 5 × first strand buffer (Invitrogen, Carlsbad, CA, USA), 2 μL 0.1 M DTT, 10 U Protector of RNase Inhibitor (Roche Diagnostics, Mannheim, Germany), 0.5 mM dNTP and 20 U Superscript III reverse transcriptase, was added to each sample. Samples were incubated at 42°C for 1 hour. Samples were diluted in DNase/RNase-free water to a working stock of 5 ng/μL of cDNA. Relative mRNA quantification was performed by real-time RT-PCR using a rotor-gene 6000 (Corbett Research, Sydney, Australia). Briefly, the reverse transcriptase was mixed with the cDNA (10 ng), gene-specific primers (200 nM) (Additional file 1: Table S1) and KAPA SYBR FAST qPCR reagent (Kapasystems, Boston, MA, USA) (5 μL), in a final volume of 10 μL. Thermal cycler parameters were as follows: 15 minutes at 95°C, followed by amplification of cDNA for 40 cycles with melting for 15 seconds at 94°C, annealing for 30 seconds at 56°C and extension for 30 seconds at 72°C. For each sample, three replicates were analyzed. Expression was normalized to GAPDH control. Fold changes were quantified as 2^-(ΔCt sample-ΔCt control)^, as described previously [43].

### Analysis of protein expression

Cells grown in 6-well plates and treated with selected compounds were washed twice with ice-cold PBS and harvested in cold RIPA buffer (Sigma-Aldrich, St. Louis, MO, USA) supplemented with 2% protease inhibitor cocktail (Roche Diagnostics, Mannheim, Germany). Extracts were centrifuged at 10,000 × g for 15 minutes at 4°C in order to remove cell debris.

Protein concentrations were determined using the Pierce® BCA protein assay kit (Thermo Scientific, Waltham, MA, USA) using BSA as protein standard. Absorbance was measured at 595 nm using a UV-max kinetic microplate reader (Molecular Devices, Devon, UK).

Equal amounts of cellular proteins (30 μg/sample) were separated by 10% sodium dodecyl sulfate-polyacrylamide gel electrophoresis (SDS-PAGE) and transferred onto polyvinylidene difluoride (PVDF) membranes (Bio-Rad Laboratories, Hercules, CA, USA) at 120 mA for 1 hour. The membranes were blocked for 1 hour in Tris-buffered saline (TBS), pH 7.4, with 0.1% Tween-20 (TBS-T) containing 10% non-fat milk. The blots were then incubated overnight at 4°C with primary antibodies against AMPKα (D63G4) (1:1,000), phospho-AMPKα (Thr172) (1:1,000), GRP78 (1:500) and β-actin (1:2,000) in TBS-T containing 5% non-fat milk. The membranes were washed 4 × 15 minutes with TBS-T and incubated with goat anti-rabbit IgG-horseradish peroxidase (1:5,000) for 2 hours. After washing the membranes with TBS-T, immunolabeling was visualized by an enhanced chemiluminescence HRP substrate (Millipore, Billerica, MA, USA) using a Fujifilm LAS-4000 detection system (Bucher Biotec, Basel, Switzerland). β-actin served as loading control of whole cell protein extracts.

### Measurement of intracellular ROS, mitochondrial mass and mitochondrial superoxide by ArrayScan® high-content imaging

Cellular ROS, mitochondrial mass and mitochondrial superoxide were detected using the specific fluorescent dyes dihydroethidium (DHE), MitoTracker®Red CMXRos and MitoSOX Red (Invitrogen, Carlsbad, CA, USA). After the treatments, DHE (10 μg/mL), MitoTracker®Red CMXRos (200 nM) or MitoSOX Red (5 μM) were added to living cells, followed by incubation for 10, 30 and 20 minutes, respectively. Cells were washed twice with PBS, fixed with 4% formaldehyde for 15 minutes and permeabilized by washing twice with 0.1% Triton X-100 in PBS. Nuclei were stained for 30 minutes with 1 μg/mL Hoechst-33342 dissolved in medium. Stained cells were visualized, images captured and the amount of fluorescence was quantified using an ArrayScan® high-content imaging system (Cellomics Inc, Pittsburgh, PA, USA). Fluorescence intensities of each dye were quantified with the cell health profiling bioapplication module. Objects were captured with the appropriate filter on 20 fields, with a total count of approximately 1,000 cells. Cells were identified using Hoechst-33342 dye and a nuclear mask was generated from images of Hoechst-stained nuclei. The values for intracellular ROS, mitochondrial mass and mitochondrial superoxide were expressed as percentage of fluorescence intensity compared with values of the control group.

### PLC activity assay

Phosphatidylcholine-specific PLC activity was determined in whole-cell lysates by using the EnzChek Direct PLC assay (Invitrogen, Carlsbad, CA, USA). Briefly, 100 μL of lysates from treated cells were incubated with 100 μL substrate working solution (glycerol-phosphoethanolamine with a dye-labeled sn-2acyl chain) for 30 minutes and fluorescence was measured at 490 nm excitation and 520 nm emission on a SpectraMax Gemini EM (Molecular Devices, Devon, UK). Enzyme activity was normalized to protein concentration of the respective cell lysate, resulting in mU/μg of protein.

### Measurement of cellular ATP content

Total ATP content was measured by a luminescence-based assay using the CellTiter-Glo® Luminescence assay kit following the manufacturer’s instruction (Promega, Madison, WI, USA). The assay buffer and substrate were equilibrated to room temperature. The buffer was transferred and gently mixed with the substrate to obtain a homogeneous solution. After cell treatment, 100 μL of the assay reagent were added into each well and the content was gently mixed under light protection on an orbital shaker to lyse the cells. After 30 minutes, the luminescence was measured on a Microplate Reader (SpectraMax GeminiEM, Molecular Devices, Devon, UK). The luminescence signals for the treated cells were normalized to the luminescence signals from the control group, which was set as 100%.

### Detection of IL-6 by enzyme-linked immunosorbent assay

IL-6 protein secretion in the culture medium of treated BV-2 cells was quantified using a commercially available ELISA kit (BD Biosciences, San Jose, CA, USA). Briefly, 96-well plates (Corning Costar 9018, Sigma-Aldrich, St. Louis, MO, USA) were coated with 50 μL of mouse IL-6 primary antibody in coating buffer and were incubated overnight at 4°C. After washing with PBS-T (PBS containing 0.05% Tween-20), the wells were blocked with 100 μL of assay diluent and incubated for 1 hour, washed, and 100 μL of samples or standards were added. After 2 hours, plates were washed and 50 μL of IL-6 detection antibody diluted 1:250 in assay diluent were added. Plates were incubated for another 1 hour prior to another wash step. The enzyme streptavidin alkaline phosphatase was diluted 1:250 in assay diluent and 50 μL were added per well, followed by incubation for 30 minutes. After washing, 100 μL of substrate solution was added and incubated for 30 minutes in the dark. The reaction was stopped with 50 μL of 1 M H_3_PO_4_ (stop solution), and absorbance was measured at 450 nm with a correction of 570 nm using a UV-max kinetic microplate reader (Molecular Devices, Devon, UK). IL-6 concentrations were calculated from the linear equation derived from the standard curve of known concentrations of purified mouse IL-6.

### Determination of intracellular Ca^2+^ concentrations

Intracellular Ca^2+^ levels were measured with the Ca^2+^-sensitive dye Fluo-4 AM. Cells were stained with 1 μg/mL Hoechst-33342 in medium for 30 minutes. Cells were then washed with HBSS containing 20 mM HEPES and equilibrated with 1 mL loading solution containing with 5 μM Fluo-4 AM, 2.5 mM probenecid and 20 mM HEPES in HBSS for 30 minutes at 37°C, followed by incubation for 30 minutes at room temperature. After loading, cells were washed twice with HBSS to remove excess fluorescent dye. Cells were treated for 1 hour with MPEP or thapsigargin, followed by the subsequent incubation with thapsigargin and MPEP, respectively, for another 1 hour. Alternatively, BV-2 cells were incubated with MPEP with or without dantrolene or xestospongin C in HBSS. The fluorescence intensity of Fluo-4 AM was measured using an ArrayScan® high-content imaging system (Cellomics Inc). Measurements of fluorescence intensity were captured with appropriate filters on 10 fields, with a total count of approximately 500 cells. Relative changes in Ca^2+^ concentrations were analyzed as a function of time and expressed as ΔF/F (in %), with F being the baseline fluorescence and ΔF the variation of fluorescence.

### Statistical analysis

The data represent mean ± SD and were analyzed using one-way ANOVA (V5.00; GraphPad Prism Software Inc., San Diego, CA, USA), followed by a Tukey’s *post hoc* test to determine the specific pairs of groups showing statistically significant differences. A *P*-value <0.05 was considered statistically significant.

## Results

### Inhibition of mGluR5 induces inflammatory mediators and cellular stress

**Figure 1:**
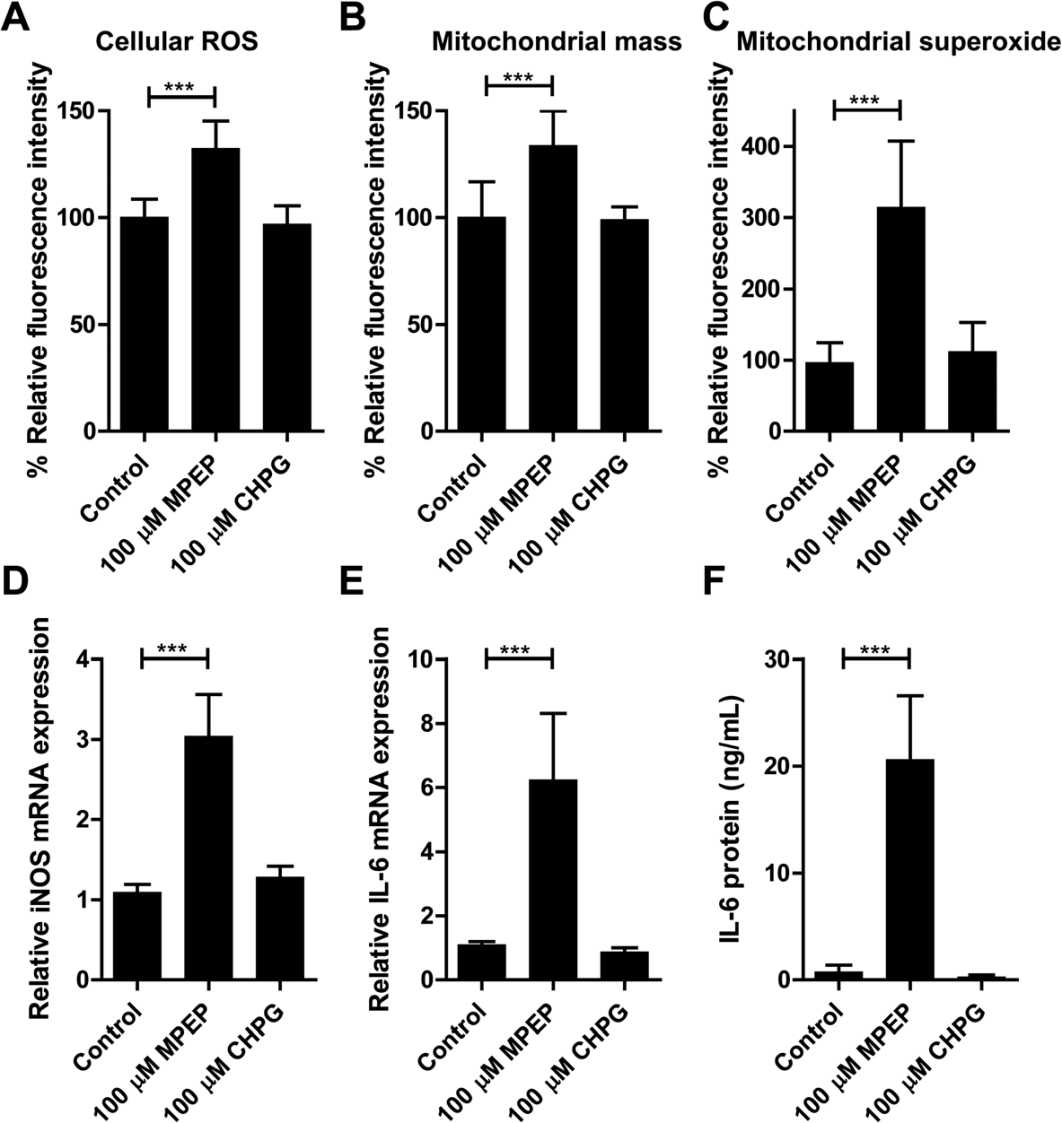
**Increased cellular stress and inflammatory mediators upon inhibition of mGluR5 in BV-2 cells.** BV-2 cells were treated for 24 hours with 100 μM of either CHPG or MPEP. **(A)** Intracellular reactive oxygen species (ROS) levels were determined using dihydroethidium (DHE). **(B)** Mitochondrial mass was measured using MitoTracker®Red CMXRos. **(C)** Mitochondrial superoxide levels were determined using MitoSOX Red. The expression of inducible nitric oxide synthase (iNOS) mRNA **(D)** and IL-6 mRNA **(E)** were measured by quantitative PCR. Results are expressed relative to the values of the house-keeping control GAPDH. **(F)** IL-6 protein levels were determined by ELISA. Results represent mean ± SD of three independent experiments. Significance was analyzed by one-way ANOVA followed by Tukey’s test. ***P <0.005. CHPG, (RS)-2-chloro-5-hydroxyphenylglycine; GAPDH, glyceraldehyde 3-phosphate dehydrogenase; MPEP, 2-methyl-6-(phenylethynyl)-pyridine.

Microglia are known to increase their production of inflammatory mediators as well as ROS and RNS upon activation. In order to investigate the role of mGluR5 on microglial activity, we treated BV-2 mouse microglia with the pharmacological mGluR5 activator CHPG and the inhibitor MPEP and assessed their impact on cellular stress markers and inflammatory mediators. After 24 hours of incubation with 100 μM of the mGluR5 antagonist MPEP, a significant increase (132 ± 5%) of intracellular ROS, detected by DHE, was observed (Figure 1A). Mitochondrial mass, measured by MitoTracker®Red CMXRos, was similarly enhanced (134 ± 6%) (Figure 1B). Mitochondrial superoxide production was assessed using MitoSOX Red, which turned out to be the most sensitive marker for oxidative stress, with an increase of 314 ± 15% upon treatment with MPEP (Figure 1C).

In addition, the mRNA expression of iNOS was determined by quantitative PCR, revealing a three-fold increase following treatment with MPEP (Figure 1D). Furthermore, MPEP enhanced the mRNA and protein levels of the inflammatory cytokine IL-6 by 6- and 29-fold (Figure 1E,F), respectively. The agonist CHPG (100 μM) had no effect on oxidative stress markers and inflammatory mediators in non-induced BV-2 cells.

### Blocking mGluR5 causes ATP depletion and results in AMPK activation

**Figure 2:**
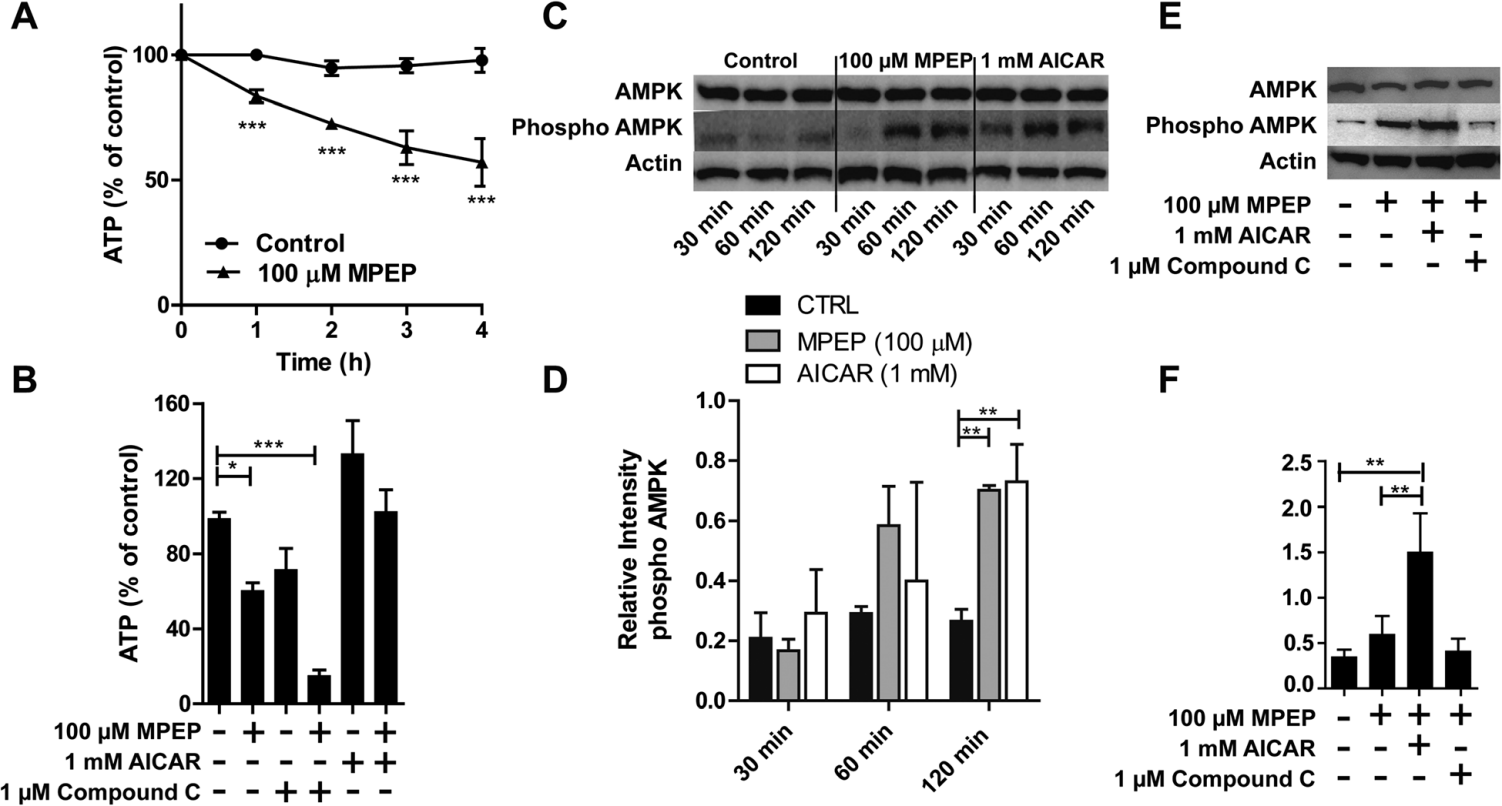
**ATP depletion and activation of AMP-dependent protein kinase (AMPK) caused by inhibition of mGluR5. (A)** BV-2 cells were incubated with 100 μM MPEP for 1, 2, 3 and 4 hours, followed by determination of cellular ATP levels. **(B)** Cells were treated for 2 hours with 100 μM MPEP, 1 μM of the AMPK inhibitor compound C, 1 mM of the AMPK activator AICAR or combinations, followed by determination of cellular ATP content. Results represent mean ± SD of three independent experiments. Significance was tested by one-way ANOVA followed by Tukey’s test. *P <0.05, ***P <0.005. **(C)** Immunoblot analyses were performed on lysates of BV-2 cells that had been treated with MPEP or AICAR (positive control) for the indicated time period. **(D)** Densitometric analysis of phospho-AMPK band normalized against β-actin. **(E)** Immunoblot analyses were performed on lysates of BV-2 cells that had been pre-incubated with either AICAR (1 mM) or compound C (1 μM) prior to incubation with MPEP for 2 hours. **(F)** Densitometric analysis of phospho-AMPK band normalized against β-actin. MPEP, 2-methyl-6-(phenylethynyl)-pyridine.

Enhanced cellular stress is often associated with changes in ATP levels. Therefore, we measured the intracellular ATP content in BV-2 cells incubated with 100 μM MPEP. A time-dependent decrease of total ATP content was observed, reaching significance as early as 1 hour after exposure to MPEP (16 ± 1% lower ATP level) (Figure 2A). As shown in Figure 2B, treatment of cells for 2 hours with 100 μM MPEP resulted in approximately 40% decreased cellular ATP levels compared with control cells. Treatment with 1 μM compound C lowered ATP levels. As expected, pretreatment of the cells with compound C potentiated the effect of MPEP to reduced ATP levels. In contrast, 1 mM of the AMPK activator AICAR tended to increase ATP levels (133 ± 7% compared with control), and pretreatment of cells with AICAR completely reversed the ATP depletion observed upon incubation with MPEP alone.

To further examine the stimulation of AMPK activation by MPEP, the phosphorylation of the AMPKα subunit was measured by immunoblotting following treatment with the chemicals indicated for 0.5, 1 and 2 hours (Figure 2C). The signal obtained for phosphorylated AMPKα was increased after 1 hour of incubation with 100 μM MPEP. The positive control (1 mM AICAR) was slightly more efficient and led to a trend increase of AMPKα phosphorylation already after 0.5 hours of incubation. AMPK phosphorylation further increased when the cells were treated with either AICAR or MPEP for 2 hours, reaching significant difference compared with control. As shown in Figure 2E,F co-incubation of BV-2 cells with MPEP and AICAR further enhanced AMPKα phosphorylation, whereas compound C was able to reverse the MPEP-induced increase of AMPKα phosphorylation.

### Increased intracellular Ca^2+^ levels following mGluR5 inhibition

Next, we tested whether the observed ATP depletion upon inhibition of mGluR5 was associated with an increased [Ca^2+^]_i_.

**Figure 3:**
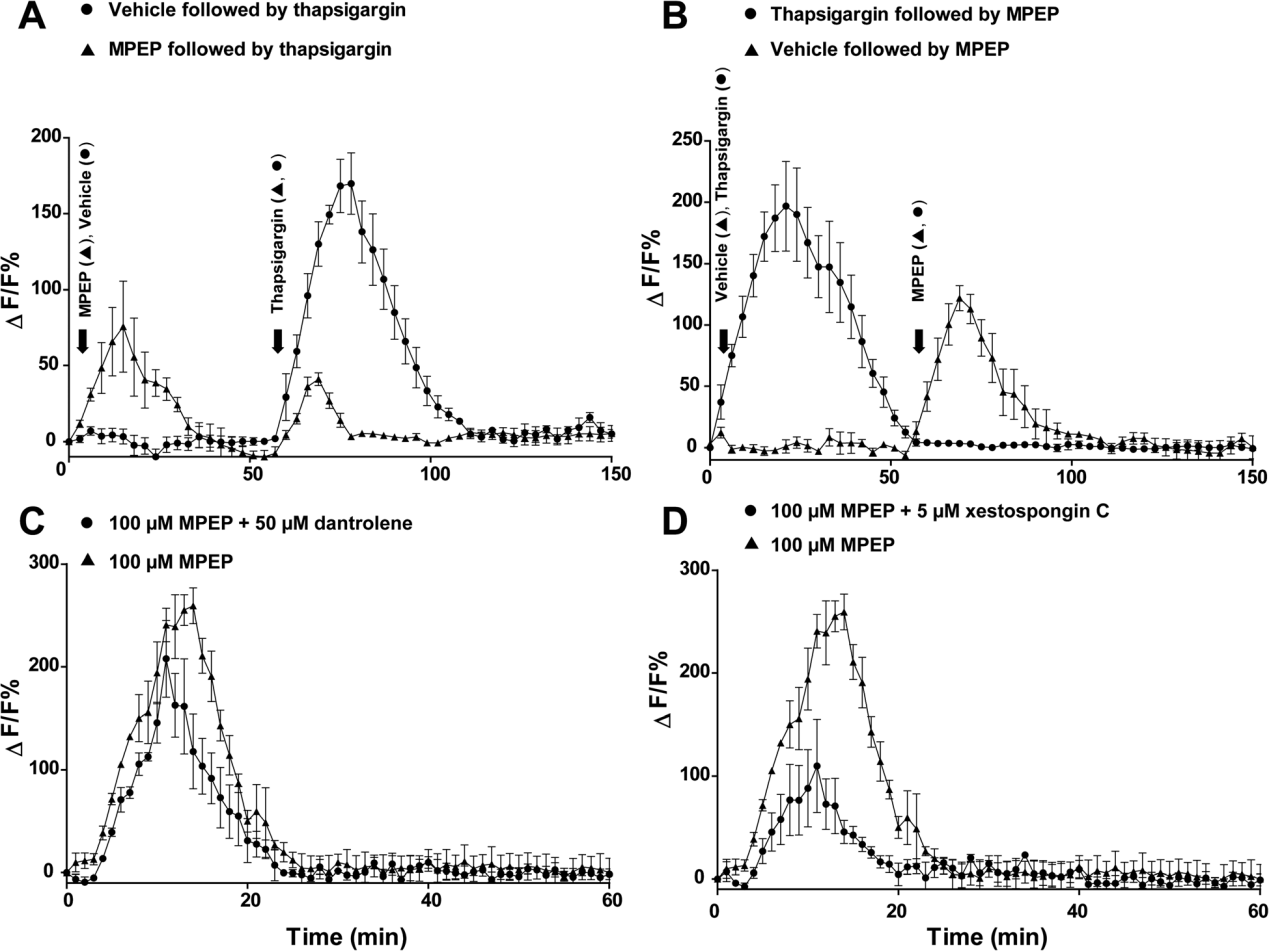
**Methyl-6-(phenylethynyl)-pyridine (MPEP) induced Ca** ^**2+**^ **release from the endoplasmic reticulum (ER).** BV-2 cells were transferred to Ca^2+^-free medium prior to the experiments and loaded with Fluo-4 AM. **(A)** Cells were incubated with 100 μM MPEP for 1 hour prior to the addition of 1 μM thapsigargin (▲). Alternatively, as a control, cells were incubated in Ca^2+^-free medium for 1 hour prior to the addition of 1 μM thapsigargin (●). Addition of thapsigargin and MPEP is indicated by an arrow. **(B)** Cells were treated with 1 μM thapsigargin for 1 hour, followed by the addition of 100 μM MPEP (●), or they were kept in Ca^2+^-free medium for 1 hour, followed by the addition of 100 μM MPEP (▲). Fluo-4 AM loaded BV-2 cells were preincubated for 1 hour with 50 μM of the ryanodine receptor (RyR) blocker dantrolene **(C)** or 5 μM of the IP_3_R blocker xestospongin C **(D)** prior to addition of 100 μM MPEP and recording of [Ca^2+^]_i_. Relative changes in Ca^2+^ concentrations using Fluo-4 AM were analyzed and expressed as ΔF/F (in %), with F being the baseline fluorescence and ΔF the variation of fluorescence. Data represent mean ± SD of three independent experiments.

The effects of MPEP on [Ca^2+^]_i_ were determined using the fluorescent indicator Fluo-4 AM. BV-2 cells kept in Ca^2+^-free medium were incubated with 100 μM MPEP and kinetic [Ca^2+^]_i_ changes were analyzed every minute. MPEP significantly increased [Ca^2+^]_i_ (Figure 3A). Subsequent addition of the ER Ca^2+^-ATPase inhibitor thapsigargin resulted in only a small increase in [Ca^2+^]_i_, compared with cells that were treated with thapsigargin alone, suggesting that the observed MPEP-induced rise in [Ca^2+^]_i_ was mainly due to Ca^2+^ release from the ER. As shown in Figure 3B, [Ca^2+^]_i_ did no longer respond to MPEP in cells that were pretreated with thapsigargin, indicating that ER Ca^2+^ stores were depleted. To provide initial evidence for the receptor involved in the Ca^2+^ efflux from the ER following exposure to MPEP, we incubated BV-2 cells with the RyR blocker dantrolene [44] or the IP_3_R blocker xestospongin C prior to addition of MPEP. While dantrolene had only a minor inhibiting effect on the increase of [Ca^2+^]_i_ by MPEP (Figure 3C), xestospongin C more effectively prevented the MPEP-induced Ca^2+^ release from the ER (Figure 3D), suggesting that IP_3_R is the major target of mGluR5-mediated signaling.

### Induction of ER-stress markers upon inhibition of mGluR5

**Figure 4:**
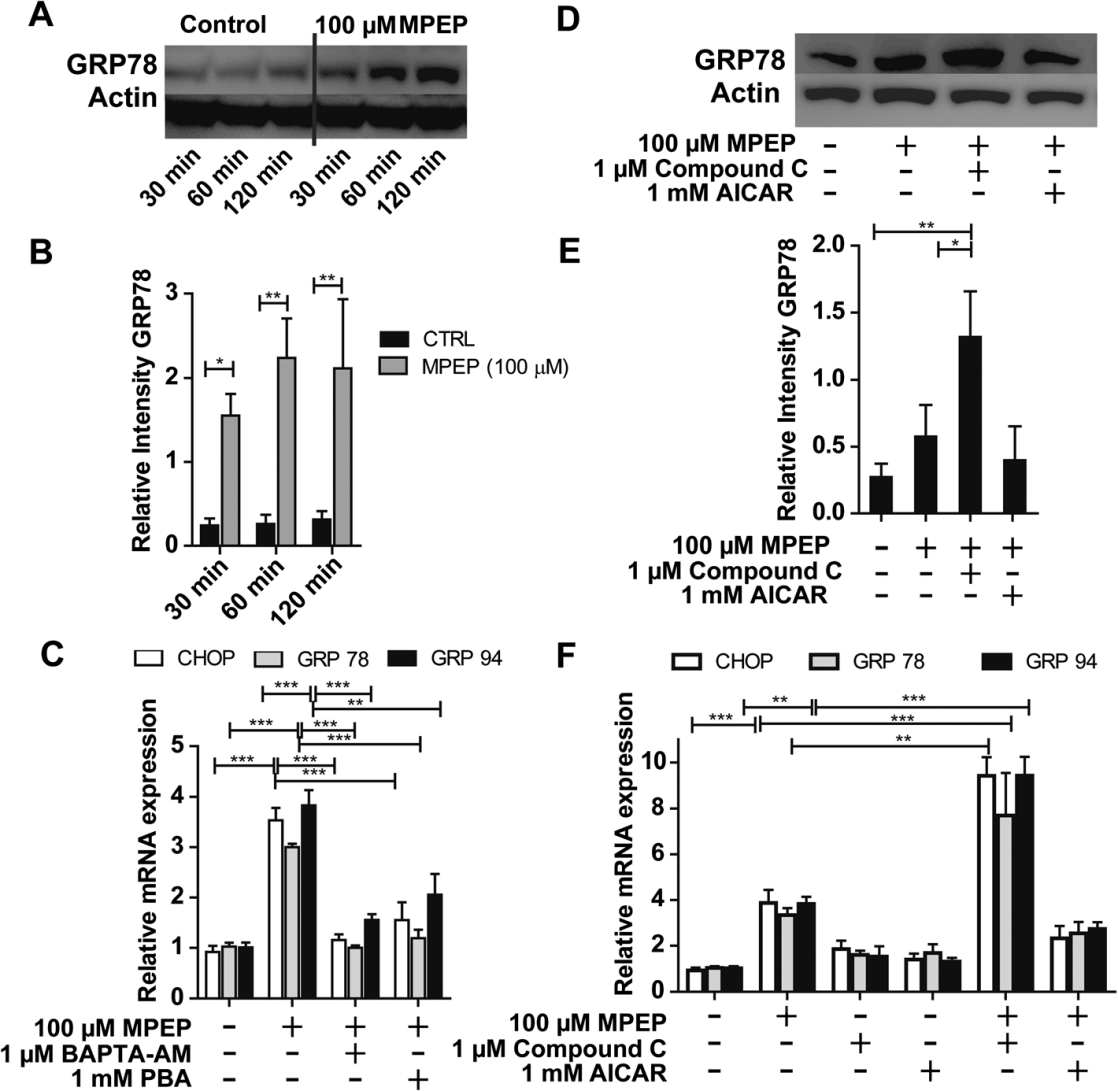
**Induction of endoplasmic reticulum (ER)-stress markers by blockage of mGluR5. (A)** Immunoblot analyses to detect GRP78 protein were performed on lysates of BV-2 cells that had been treated with 100 μM MPEP for the indicated time period. β-actin served as a loading control. **(B)** Densitometric analysis of GRP78 band normalized against β-actin. **(C)** Cells were preincubated for 1 hour with 1 μM BAPTA-AM or 1 mM sodium phenylbutyrate (PBA), followed by further incubation with 100 μM MPEP for 24 hours. Expression of CHOP, GRP78 and GRP94 mRNA were measured by quantitative PCR. **(D)** Cells were pre-exposed for 1 hour with 1 mM AICAR or 1 μM compound C, followed by a further incubation with 100 μM MPEP for 2 hours and GRP78 protein levels were analyzed by immunoblotting. **(E)** Densitometric analysis of GRP78 band normalized against β-actin. **(F)** Cells were pre-exposed for 1 hour with 1 mM AICAR or 1 μM compound C, followed by a further incubation with 100 μM MPEP. Expression of CHOP, GRP78 and GRP94 mRNA were measured by quantitative PCR and mRNA expression levels were normalized to GAPDH control. Results represent mean ± SD of three independent experiments. Significance was analyzed by one-way ANOVA followed by Tukey’s tests. **P <0.01; ***P <0.005. CHOP, C/EBP homologous protein; GAPDH, glyceraldehyde 3-phosphate dehydrogenase; MPEP, 2-methyl-6-(phenylethynyl)-pyridine.

Disturbances in Ca^2+^ homeostasis can trigger ER-stress; therefore we tested whether inhibition of mGluR5 by MPEP induces ER-stress in BV-2 cells. MPEP treatment resulted in a time-dependent increase in GRP78 protein expression (Figure 4A,B). Furthermore, the mRNA expression levels of the ER-stress markers CHOP, GRP78 and GRP94 were significantly increased after exposure to MPEP for 24 hours (3.5-fold, 3.0-fold and 3.8-fold compared with control, respectively) (Figure 4C). To further study the impact of MPEP on ER-stress, we pretreated cells for 1 hour with 1 mM of the chemical chaperone 4-phenylbutyrate (4-PBA), followed by incubation with MPEP for 24 hours. The MPEP-induced increase in the mRNA expression of the major ER-stress markers was almost fully prevented by 4-PBA (Figure 4C). To test whether the rise in [Ca^2+^]_i_ contributes to the increased expression of the ER-stress markers, we pretreated BV-2 cells for 1 hour with 1 μM of the Ca^2+^ chelator BAPTA-AM prior to further incubation in the presence of 100 μM MPEP for 24 hours. Pretreatment of cells with BAPTA-AM abolished the induction of the expression of the three ER-stress markers, indicating that the rise in [Ca^2+^]_i_ is a critical step in the cellular stress response to mGluR5 inhibition.

To evaluate whether the effect of MPEP on ER-stress involves AMPK, we examined the effect of AICAR and compound C on GRP78 protein expression. Cells were pretreated with either AICAR (1 mM) or compound C (1 μM) for 1 hour, followed by incubation with MPEP (100 μM) for 2 hours. AICAR or compound C treatment alone did not significantly alter GRP78 protein levels (data not shown). However, compound C potentiated the MPEP-mediated induction of GRP78 expression (Figure 4D,E). In contrast, activation of AMPK activity by AICAR tended to reduce the MPEP-dependent induction of GRP78 protein expression. Furthermore, we determined the impact of pretreatment of the cells with either AICAR (1 mM) or compound C (1 μM) for 1 hour on the effect of 100 μM MPEP for 24 hours on the mRNA levels of CHOP, GRP78 and GRP94 (Figure 4F). Compound C partially prevented the increase in CHOP, GRP78 and GRP94 mRNA expression levels upon exposure to MPEP, whereas AICAR potentiated the MPEP-stimulated mRNA expression of the ER-stress markers. These findings strongly suggest that AMPK is involved in the MPEP-mediated effects on ER-stress markers in BV-2 cells.

### Ca^2+^ release upon inhibition of mGluR5 is dependent on PLC

**Figure 5:**
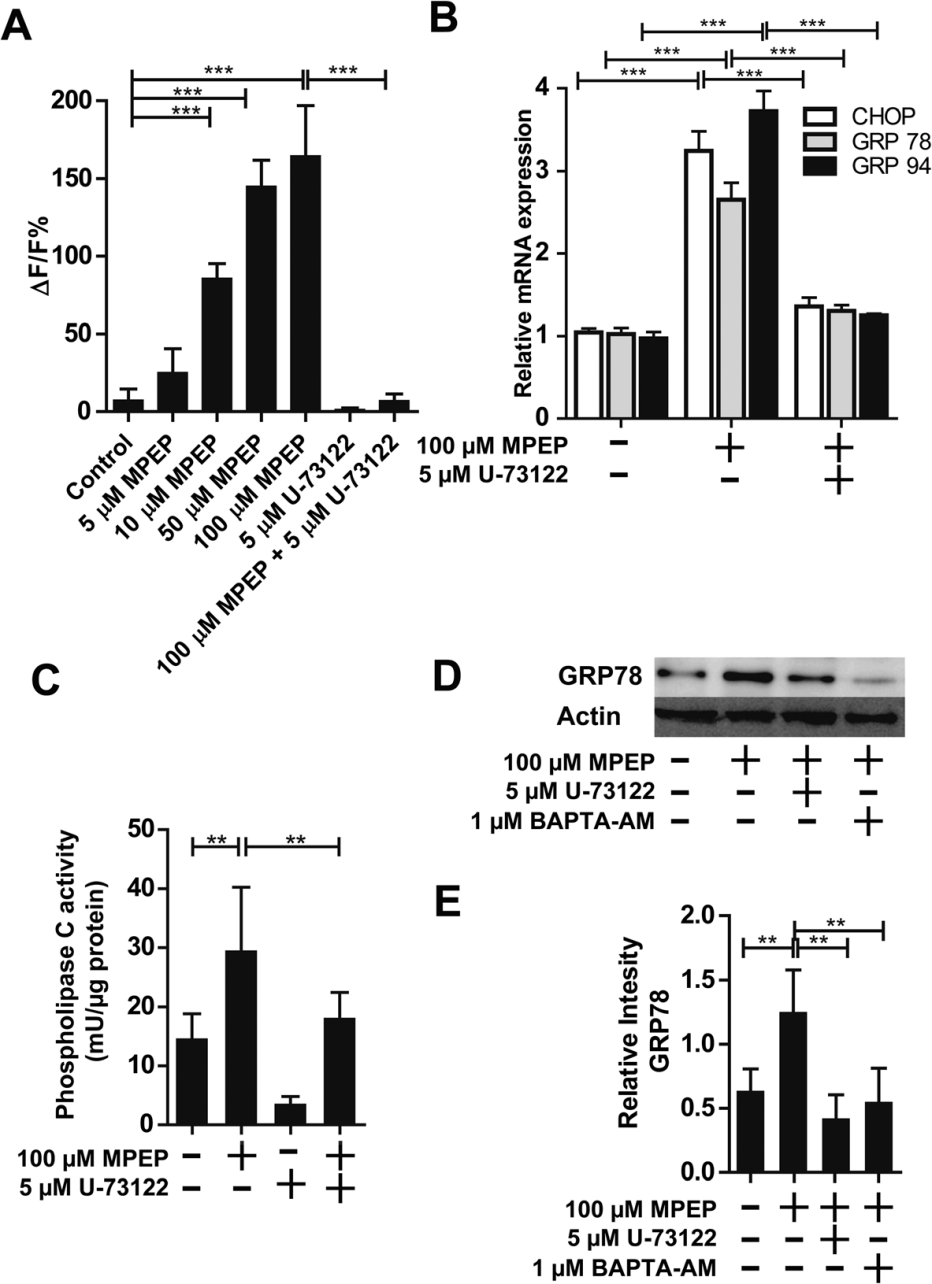
**Activation of phospholipase C (PLC) upon inhibition of mGluR5. (A)** Intracellular Ca^2+^ concentrations were determined by measuring the fluorescence intensity of the fluorochrome Fluo-4 AM. BV-2 cells were pretreated for 1 hour with 5 μM U-73122 prior to the addition of 100 μM MPEP. Relative changes in the Ca^2+^ concentrations detected by Fluo-4 AM were analyzed after 15 minutes. Results are expressed as ΔF/F (in %), with F being the baseline fluorescence and ΔF the variation of fluorescence. Data represent the mean ± SD. **(B)** Cells were incubated for 24 hours with 100 μM MPEP in the presence or absence of 5 μM of the phospholipase C inhibitor U-73122, followed by determination of mRNA expression of CHOP, GRP78 and GRP95 by quantitative PCR. Results were normalized to GAPDH mRNA control. Results represent the mean ± SD from three independent experiments, each performed in triplicate. **(C)**, BV-2 cells were incubated with 100 μM MPEP for 2 hours with or without pretreatment for 1 hour with 5 μM U-73122, followed by determination of PLC activity. PLC activity (mU/μg total protein) is given as the mean ± SD from three independent experiments. **(D)** BV-2 cells were incubated with 100 μM MPEP in the absence or presence of 5 μM PLC inhibitor U-73122 or 1 μM Ca^2+^ chelator BAPTA-AM, followed by the determination of GRP78 protein expression by immunoblotting. **(E)** Densitometric analysis of GRP78 band normalized against β-actin. **P <0.01; ***P <0.001. CHOP, C/EBP homologous protein; GAPDH, glyceraldehyde 3-phosphate dehydrogenase; MPEP, 2-methyl-6-(phenylethynyl)-pyridine.

Activation of PLC produces IP_3_, which activates IP_3_R, thereby inducing Ca^2+^ release from the ER in a variety of cells. Therefore, we investigated a possible role of PLC in the MPEP-dependent Ca^2+^ release from the ER. As shown in Figure 5A, MPEP increased [Ca^2+^]_i_ in a concentration-dependent manner. Importantly, pretreatment of cells with 5 μM of the PLC inhibitor U-73122 completely abolished the effect of 100 μM MPEP. Furthermore, the MPEP-dependent induction of CHOP, GRP78 and GRP96 mRNA expression, measured after 24 hours, was also prevented by cotreatment of the cells with U-73122 (Figure 5B). Next, we investigated whether MPEP leads to enhanced PLC activity. Total PLC activity was significantly increased in cells treated with MPEP for 2 hours compared with control (Figure 5C). The MPEP-induced increase in total PLC activity was completely blocked by pretreatment with U-73122 for 1 hour. U-73122 also significantly decreased basal PLC activity in BV-2 cells. In addition, inhibition of PLC by U-73122 reduced the MPEP-induced up-regulation of GRP78 protein expression (Figure 5D,E). Cotreatment of the cells with BAPTA-AM also abolished the effect of MPEP, indicating that Ca^2+^ is directly involved in the MPEP-dependent induction of ER-stress markers. These results revealed a role for PLC in the MPEP-induced Ca^2+^ elevation and ER-stress.

### Pertussis toxin-sensitive G-protein coupled receptors are involved in the mGluR5-mediated effects

**Figure 6:**
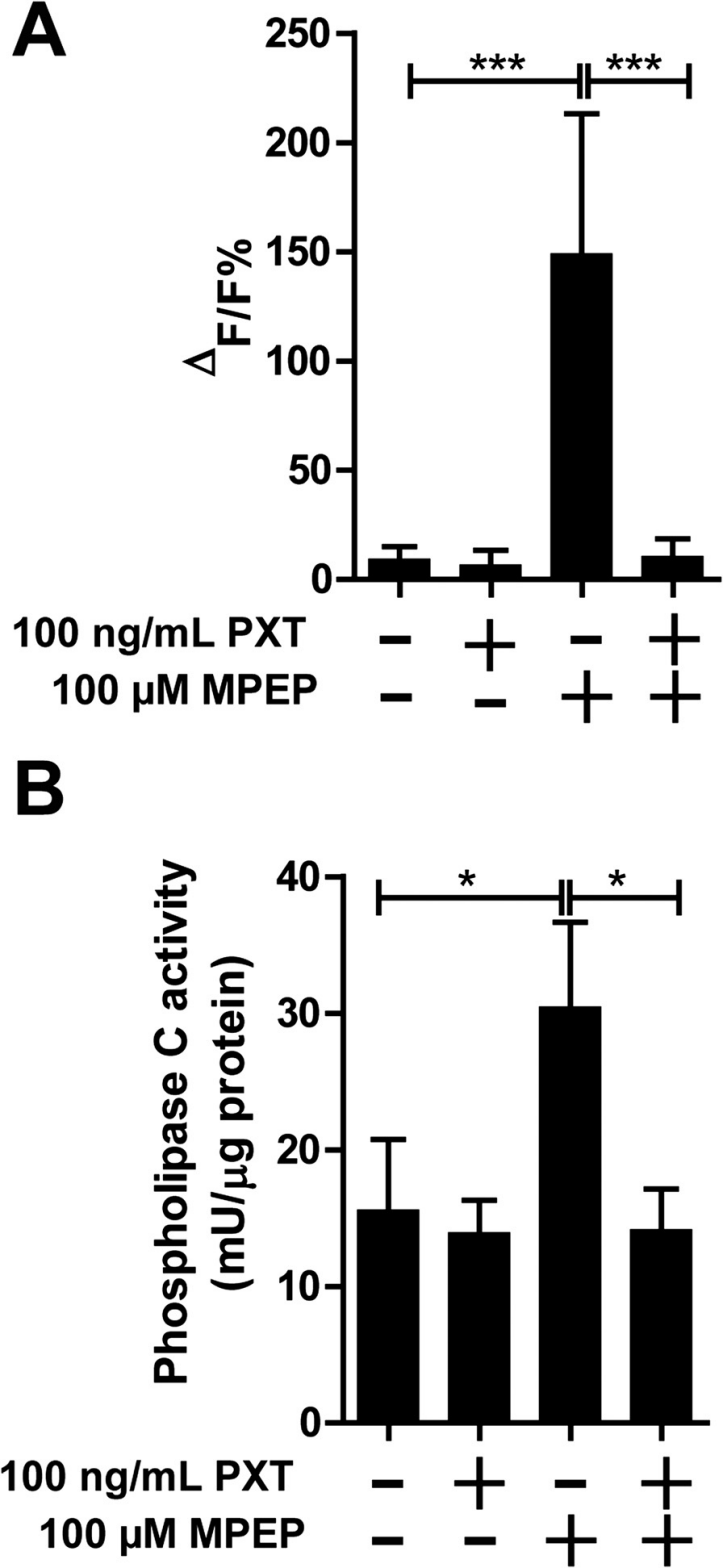
**Involvement of pertussis toxin-sensitive G-proteins in the mGluR5-mediated effects. (A)** BV-2 cells were pretreated for 6 hours with 100 ng/mL pertussis toxin (PTX) prior to loading with the fluorochrome Fluo-4 AM and the addition of 100 μM MPEP. Intracellular Ca^2+^ concentrations were determined by detecting the fluorescence intensity of Fluo-4 AM. Relative changes of the Ca^2+^ concentrations were analyzed after 15 minutes. Results are expressed as ΔF/F (in %), with F being the baseline fluorescence and ΔF the variation of fluorescence. **(B)** BV-2 cells were pretreated for 6 hours with 100 ng/mL PTX, followed by treatment with 100 μM MPEP for 2 hours. Phospholipase C (PLC) activity was adjusted to total protein content (mU/μg total protein). Results represent mean ± SD of three independent experiments and significance was analyzed by one-way ANOVA followed by Tukey’s test. *P <0.05, ***P <0.005. MPEP, 2-methyl-6-(phenylethynyl)-pyridine.

Several studies have demonstrated that G-protein coupled receptors may exert their effects through activation of PLC, which hydrolyzes phospholipids in cell membranes to IP_3_ and diacylglycerol. To investigate the [Ca^2+^]_i_ responses to MPEP, we pretreated cells for 6 hours with 100 ng/mL pertussis toxin (PTX), a specific inhibitor of Gi/o-type G-proteins. As shown in Figure 6A, pretreatment of cells with PTX completely abolished the MPEP-induced increase in [Ca^2+^]_i_, suggesting the involvement of Gi/o-proteins in [Ca^2+^]_i_ responses to MPEP. Pretreatment of BV-2 cells with PTX prevented the MPEP-dependent activation of PLC, while PTX alone did not affect basal activity of PLC (Figure 6B). These results suggest that MPEP utilizes a PTX-sensitive G-protein in the activation of PLC, which leads to enhanced [Ca^2+^]_i_ and activation of ER-stress markers. To further investigate the downstream effects of MPEP after PLC activation and increase in [Ca^2+^]_i_, we investigated whether PTX might suppress the MPEP-dependent induction of GRP78 expression. Upon pretreatment of cells for 6 hours with PTX a trend decrease in MPEP-induced GRP78 expression was observed by Western blot analysis; however, the results did not reach significance (data not shown).

### Induction of cellular stress and inflammatory mediators upon mGluR5 inhibition can be prevented by interfering with intracellular signaling

**Figure 7:**
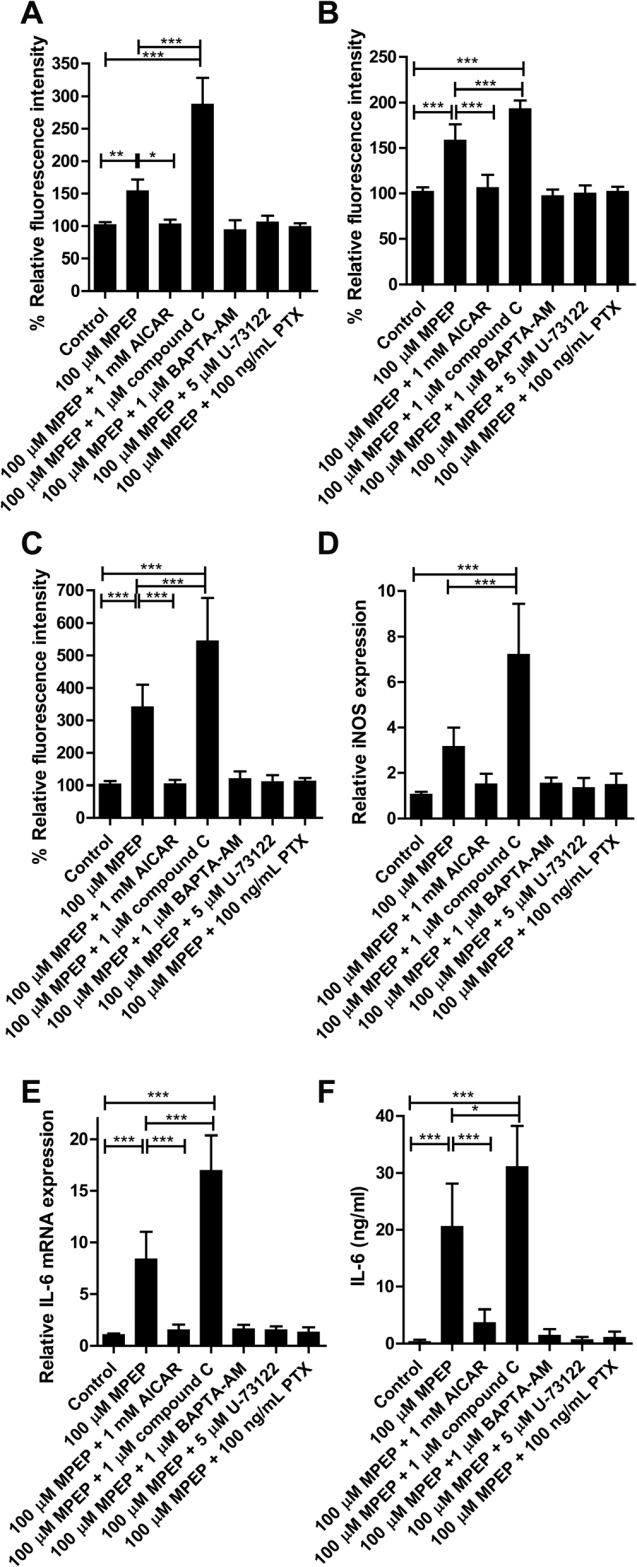
**Prevention of cellular stress and inflammatory mediators by interference with intracellular signaling.** BV-2 cells were pretreated for 1 hour with 1 μM compound C, 1 μM BAPTA-AM or 5 μM U-73122, or for 6 hours with pertussis toxin (PTX) (100 ng/mL) prior to incubation with 100 μM MPEP for 24 hours. **(A)** Intracellular reactive oxygen species (ROS) levels were determined using dihydroethidium (DHE). **(B)** Mitochondrial mass was measured using MitoTracker®Red CMXRos. **(C)** Mitochondrial superoxide levels were detected using MitoSOX Red. The mRNA expression of inducible oxygen species (iNOS) **(D)** and IL-6 **(E)** were determined by quantitative PCR and values were normalized to GAPDH mRNA control. **(F)** IL-6 protein expression was measured by ELISA. Results represent the mean ± SD of at least three independent experiments. Significance was tested by one-way ANOVA followed by Tukey’s test. *P <0.05, **P <0.01; ***P <0.005. GAPDH, glyceraldehyde 3-phosphate dehydrogenase; MPEP, 2-methyl-6-(phenylethynyl)-pyridine.

In order to further study the impact of the intracellular signaling pathways that were shown to be influenced by mGluR5 above, we pretreated BV-2 cells with the various modulators and measured the effects of MPEP exposure on the regulation of cellular stress and inflammatory mediators. The levels of intracellular ROS, mitochondrial mass, mitochondrial superoxide production, iNOS mRNA as well as IL-6 mRNA and protein were determined. Pretreatment of cells with AICAR reversed the effects of MPEP on cellular stress parameters and cytokine expression, whereas compound C potentiated them (Figure 7). BAPTA-AM, U-73122 and PTX completely abolished the MPEP-dependent induction of ROS production and inflammatory cytokine expression. Together, these data suggest that blockage of mGluR5 by MPEP leads to the activation of PTX-sensitive G-proteins that causes activation of PLC, with subsequent Ca^2+^ release from the ER, thereby producing cellular stress and enhancing the expression of inflammatory cytokines.

## Discussion

Several reports have shown that mGluR5 exerts neuroprotective effects in microglia cells by modulating oxidative stress and inflammatory cytokine release both *in vitro* and *in vivo*. It has been shown that the mGluR5 agonist CHPG diminished NF-κB activation, NADPH oxidase activity and ROS production in lipopolysaccharide (LPS)-stimulated microglia and improved functional recovery after traumatic brain injury [7,8,12]. Delayed CHPG administration suppressed the activation of microglia and chronic neuroinflammation and decreased the hippocampal neuronal loss and lesion progression following experimental traumatic brain injury in mice [9]. Byrnes *et al*. showed that in primary microglia from mGluR5-deficient mice CHPG was no longer able to decrease the LPS-induced inflammation and oxidative stress, indicating a key role for mGluR5 in the modulation of microglial activation [10]. Furthermore, they provided evidence for a role of PLC, PKC and Ca^2+^ in the mGluR5-mediated effects. In contrast to its activation, the possible consequences of the inhibition of mGluR5 by xenobiotics on cellular stress parameters and inflammatory mediators in microglia remains less well understood.

**Figure 8:**
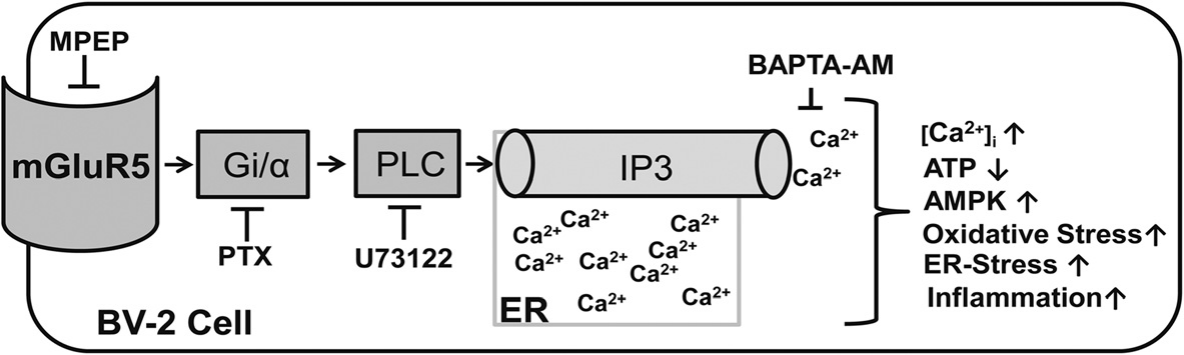
**Schematic presentation of signaling mechanisms and cellular outcomes following mGluR5 blockade.**

In the present study, we used BV-2 mouse microglia cells, which show highly overlapping gene expression profiles with primary mouse microglia and therefore represent a valuable model system for primary mouse microglia [41], to investigate the effects of the mGluR5 blocker MPEP on cellular stress parameters, inflammatory mediators and the underlying signaling pathways. Although MPEP is widely used to modulate the mGluR5 receptor it has been reported that the compound lacks sensitivity against mGluR5, leading to off-target effects while antagonizing other receptors. Thus, it has been shown that MPEP exerts neuroprotective effects at high concentrations, mediated by inhibition of the N-methyl-D-aspartate receptor (NMDA) [45]. MPEP, at the concentrations used in the present study, may have off-target effects *in vivo*. Specifically, MPEP has been shown to antagonize the mGluR1 and NR2B receptors [46] and positively regulate the mGluR4 receptor [47]. BV-2 cells express high protein levels of mGluR5 and very low levels of mGluR1 [13]. Additionally, evidence shows that the effect of MPEP on the NR2B and mGluR4 receptor would be neuroprotective [47,48]. Since our results show that MPEP induces cellular stress, we are confident that MPEP is targeting mGluR5; however, contribution of other affected targets to the observed results cannot be excluded. More selective antagonists of mGluR5 receptors such as the recently developed 3-[(2-methyl-1,3-thiazol-4-yl) ethynyl] pyridine (MTEP) might be used to address possible off-target effects. Exposure of BV-2 cells to MPEP increased total and mitochondrial ROS production, caused mitochondrial swelling and enhanced the expression of iNOS as well as the expression and release of IL-6 (Figure 8). CHPG completely reversed the effects of MPEP, indicating that the MPEP-induced oxidative stress and up-regulation of cytokines was mediated by mGluR5 inactivation (data not shown). These results are consistent with previous studies reporting a protective role of microglial mGluR5 activation on neuronal cells and in preventing brain damage [9–12,49].

On the other side, some studies provided evidence for neuroprotective effects of mGluR5 inhibition by MPEP. A prolonged exposure of spinal cord motor neurons to cultures enriched in reactive astrocytes to MPEP reduced AMPA-mediated toxicity and cobalt uptake in motor neurons [50]. The protective effect of MPEP was absent in cultures with low astrocyte numbers, suggesting that blockade of mGluR5 in astrocytes was responsible for the observed effects. Thus, mGluR5 likely exerts differential effects in microglia and astrocytes. Another study showed a neuroprotective effect of MPEP in a rodent 6-hydroxydopamine Parkinson’s disease model [51]. Their results suggested that enhanced glutaminergic activity to the substantia nigra pars compacta may contribute to the progression of nigral dopaminergic neuronal degeneration and that mGluR5 blockers might delay disease progression. The exact mode of action, however, remained unclear.

To begin to understand why mGluR5 blockade results in enhanced cellular stress in microglia, we investigated the mGluR5-mediated signaling pathway in BV-2 cells upon treatment with MPEP. Our results revealed a rapid increase in [Ca^2+^]_i_, with peak concentrations reached 15 minutes after addition of MPEP and return to baseline after 40 minutes. The fact that pre-exposure of cells to thapsigargin completely abolished the MPEP-induced increase in [Ca^2+^]_i_, and *vice versa* pre-incubation with MPEP reduced thapsigargin-induced [Ca^2+^]_i_ increase, indicated that Ca^2+^ was released from the ER. Furthermore, the IP_3_R blocker xestospongin C effectively prevented the MPEP-induced Ca^2+^ release from the ER, whereas the RyR antagonist dantrolene was less effective, suggesting a major role for IP_3_R in the mGluR5-dependent signaling pathway.

Changes in [Ca^2+^]_i_ represent a major signaling pathway in microglia. Exposure of microglia to LPS leads to chronically elevated [Ca^2+^]_i_ and it was shown that the Ca^2+^ chelator BAPTA-AM could prevent the LPS-induced release of NO and the cytokines TNF-α, IL-6 and IL-12 [52]. Importantly, blockade of IP_3_R by xestospongin C has been shown to abolish the production of TNF-α and IL-6 in activated microglia [53].

Impaired microglial Ca^2+^-mediated signal transduction was observed under pathological conditions. Exposure to amyloid-β increased [Ca^2+^]_i_ in cultured microglia, and basal [Ca^2+^]_i_ was higher in microglia isolated from the brain of Alzheimer’s patients compared with microglia from non-demented individuals [54,55]. Increased [Ca^2+^]_i_ may act directly on the mitochondrial membrane, leading to enhanced ROS production [56,57]. Furthermore, a Ca^2+^ overload decreases mitochondrial function by triggering the opening of the mitochondrial membrane permeability transition pore, resulting in mitochondrial membrane depolarization and swelling, enhanced ROS generation and decreased ATP production [58–60].

Mitochondrial dysfunction and ATP depletion have been associated with increased oxidative stress and neuroinflammation [61–63]. The rapid ATP depletion observed in the present study in BV-2 cells following incubation with MPEP could be caused by excessive ATP consumption by ER Ca^2+^-ATPases attempting to lower [Ca^2+^]_i_ by Ca^2+^ reuptake into the ER and/or mitochondrial dysfunction and impaired ATP production. The MPEP-dependent ATP depletion was ameliorated by pretreatment of cells with the AMPK inducer AICAR, indicating an involvement of AMPK in the modulation of the mGluR5-mediated effects. The depletion of ATP is likely to occur prior to the induction of AMPKα phosphorylation, in line with the role of AMPK in restoring cellular ATP levels [64]. Studies in BV-2 microglia and in glial cells have shown that the pharmacological activation of AMPK suppresses the pro-inflammatory responses [65,66]. On the other side, AMPK activation was found to enhance ROS production in microglia and in pancreatic beta-cells exposed to high glucose levels [67–69], and some of the observations may depend on the specific conditions of the respective study. Nevertheless, the results of the present study provide, to our knowledge, the first evidence for an association between mGluR5 inhibition and subsequent ATP depletion and AMPK activation in microglia.

The excessive Ca^2+^ release from the ER may be responsible for the observed ATP depletion. It may further lead to the depletion of ER luminal Ca^2+^ stores, which is believed to trigger ER-stress and activate the UPR pathway, including the induction of the expression of ER chaperones such as CHOP, GRP78 and GRP96 [35]. In this study, we observed that the MPEP-induced expression of CHOP, GRP78 and GRP96 was attenuated by the Ca^2+^ chelator BAPTA-AM in BV-2 cells, suggesting that the ER-stress, caused upon mGluR5 inhibition, was dependent on increased [Ca^2+^]_i_.

Impaired AMPK signaling has been associated with ER-stress in different cell types [36,38,70]. We found that the MPEP-mediated induction of ER-stress markers was potentiated by the AMPK inhibitor compound C and diminished by the activator AICAR. Importantly, pretreatment of BV-2 cells with either compound C or AICAR did not affect the MPEP-dependent increase in [Ca^2+^]_i_ (data not shown), suggesting that the Ca^2+^ release from the ER precedes ATP depletion and AMPK activation. The protective effects of pretreatment of cells with AICAR may be explained by an increased cytoplasmic ATP concentration, which may facilitate energy dependent transport of Ca^2+^ back into the ER following mGluR5 blockade.

The MPEP-dependent increase in [Ca^2+^]_i_ and subsequent induction of ER-stress markers and mitochondrial dysfunction involved the activation of PLC and could be prevented by pretreatment of BV-2 cells with the PLC inhibitor U-73122. Importantly, an increased PLC activity could be measured following incubation of microglia with MPEP, suggesting that mGluR5 inhibition caused cellular stress via the excessive activation of the PLC-IP_3_ pathway. It was reported that the mGluR5 agonist CHPG reduced the LPS-dependent inflammatory response and activated PLC, thereby increasing [Ca^2+^]_i_ in microglia [10]. Thus, a tight control of the balance of mGluR5 signaling is important to avoid cellular stress. Additionally, mGluR5 might cross regulate toll-like receptors, thereby resulting in a muted inflammatory response.

Classically, the activation of PLC resulting in the synthesis of IP_3_ and the release of Ca^2+^ from the ER was associated with Gq-coupled receptors [39]. In the present study, the MPEP-induced increase in [Ca^2+^]_i_ was attenuated by the Gi-protein inhibitor PTX, the PLC inhibitor U-73122 and the IP_3_R antagonist xestospongin C, thus suggesting crosstalk between mGluR5 receptors and Gi-protein subunits to modulate PLC and induce IP_3_R-mediated Ca^2+^ release from the ER. Several studies in a variety of cells and tissues demonstrated an increase in [Ca^2+^]_i_ following stimulation of Gi-coupled receptors [39,71,72]. For instance, treatment of natural killer cells with PTX diminished intracellular Ca^2+^ influx and chemotaxis induced by oxidized lipids and lysophosphatidylcholine [72]. Further, PTX completely blocked the Ca^2+^ responses induced by the endogenous μ-opioid receptor agonist endomorphin-1 in astrocytes [73].

## Conclusions

The present study demonstrates for the first time that antagonism of mGluR5 in BV-2 microglia results in increased cellular stress with mitochondrial dysfunction and ER-stress caused by an activation of PTX-sensitive Gi-proteins and subsequent PLC activation and IP_3_-dependent Ca^2+^ release from the ER. The increase in [Ca^2+^]_i_ upon inhibition of mGluR5 was followed by a depletion of cellular ATP and activation of AMPK, resulting in the induction of pro-inflammatory mediators. The results suggest that xenobiotics inhibiting mGluR5 disrupt Ca^2+^ signaling and redox regulation in microglia, thereby promoting neuroinflammation.

## Additional file

Additional file 1: Table S1. Oligonucleotide primers for real-time PCR.

## Abbreviations

[Ca^2+^]_i_: intracellular free Ca^2+^
AMPK: AMP-dependent protein kinase
CHOP: transcriptional factor C/EBP homologous protein
CHPG: (RS)-2-chloro-5-hydroxyphenylglycine
DEPC: diethylpyrocarbonate
DHE: dihydroethidium
DMEM: Dulbecco’s modified Eagle’s medium
ELISA: enzyme-linked immunosorbent assay
ER: endoplasmic reticulum
ERK1/2: extracellular signal-regulated protein kinases 1/2
FBS: fetal bovine serum
GAPDH: glyceraldehyde 3-phosphate dehydrogenase
GPCR: G-protein-coupled receptor
GRP78: glucose-regulated protein 78
GRP94: glucose-regulated protein 94
HBSS: Hank’s balanced salt solution
HEPES: 4-(2-hydroxyethyl)-1-piperazineethanesulfonic acid
IL-6: interleukin-6
iNOS: inducible nitric oxide synthase
IP_3_: inositol triphosphate
IP_3_R: inositol triphosphate-induced receptor
LPS: lipopolysaccharide
MAPKs: mitogen-activated protein kinases
mGluR5: metabotropic glutamate receptor 5
MPEP: 2-methyl-6-(phenylethynyl)-pyridine
NEAA: non-essential amino acids
NF-κB: nuclear factor kappaB
NO: nitric oxide
NOX-2: NADPH oxidase type 2
PBA: sodium phenylbutyrate
PBS: phosphate-buffered saline
PCR: polymerase chain reaction
PDVF: polyvinylidene difluoride
PERK: PKR-like ER kinase
PKC: protein kinase C
PLC: phospholipase C
PTX: pertussis toxin
RIPA: radio immunoprecipitation assay
ROS: reactive oxygen species
RNS: reactive nitrogen species
RyR: ryanodine receptor
SDS-PAGE: sodium dodecyl sulfate-polyacrylamide gel electrophoresis
TBS: Tris-buffered saline
TBS-T: Tris-buffered saline with Tween
TNF: tumor necrosis factor
UPR: unfolded protein response
