# Supplementary material for: Inhibition of metabotropic glutamate receptor 5 induces cellular stress through pertussis toxin-sensitive Gi-proteins in murine BV-2 microglia cells
Source: J Neuroinflammation. 2014 Nov 19;11:190. doi: 10.1186/s12974-014-0190-7 (PMC4240888; doi:10.1186/s12974-014-0190-7)
Supplement: Additional file 1: Table S1. — Oligonucleotide primers for real-time PCR. [file 12974_2014_190_MOESM1_ESM.doc]

**Additional file 1: Table S1**

Oligonucleotide primers for real-time PCR

| **Genes** | | **Primers** | **Sequences** |
| --- | --- | --- | --- |
| GAPDH (mouse) | | forward | 5’-CTCGTGGAGTCTACTGGTGT-3’ |
|  |  | reverse | 5’-GTCATCATACTTGGCAGGTT-3’ |
| IL-6 (mouse) | | forward | 5’-GGAGGCTTAATTACACATGTT-3’ |
|  |  | reverse | 5’-TGATTTCAAGATGAATTGGAT-3’ |
| TNF-α (mouse) | forward | | 5’-TTCTGTCTACTGAACTTCGG-3’ |
|  | reverse | | 5’-GTATGAGATAGCAAATCGGC-3’ |
| iNOS (mouse) | forward | | 5’-ATGAGGTACTCAGCGTGCTCCAC-3’ |
|  | reverse | | 5’-CCACAATAGTACAATACTACTTGG-3’ |
